# Facile protein conjugation of platinum for light-activated cytotoxic payload release†

**DOI:** 10.1039/d1cc02722k

**Published:** 2021-07-12

**Authors:** Cinzia Imberti, Frederik Lermyte, Emily P. Friar, Peter B. O'Connor, Peter J. Sadler

**Affiliations:** Department of Chemistry, University of Warwick, Gibbet Hill Road Coventry CV4 7AL UK cinzia.imberti@warwick.ac.uk P.J.Sadler@warwick.ac.uk; Department of Chemistry, Technical University of Darmstadt, Alarich-Weiss-Strasse 4 Darmstadt 64287 Germany

## Abstract

The novel Pt(iv) complex *trans*,*trans*-[Pt(N_3_)_2_(Py)_2_(OH)(OCO-(PEG)_2_-NHCSNH-Ph-NCS)] (Pt4) conjugates to the side chain of lysine amino acids in proteins under mild conditions. Reaction with myoglobin generated a bioconjugate that was stable in the dark, but released a Pt(iv) prodrug upon visible light irradiation. A similar procedure was used to conjugate Pt4 to the antibody trastuzumab, resulting in the first photoactivatable Pt(iv)-antibody conjugate, demonstrating potential for highly selective cancer phototherapy.

## Abstract 2

This platinum anticancer complex can be conjugated to proteins, including antibodies, under mild conditions and activated by visible light, providing a basis for highly selective cancer phototherapy.

## Graphical abstract

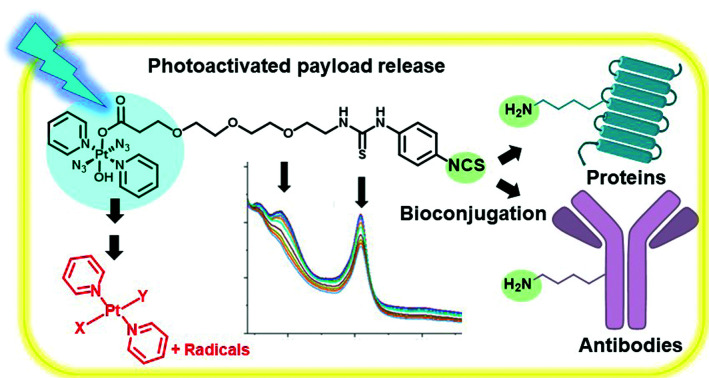

Despite their widespread clinical use, platinum(ii) anticancer drugs often present severe side effects, which critically affect patients’ quality of life. The use of relatively inert Pt(iv) prodrugs that can undergo intracellular reduction to cytotoxic Pt(ii) species in cancer cells has been widely investigated to minimise systemic toxicity.^1^ However, non-tumour specific reduction limits the benefits of these agents. No Pt(iv) chemotherapeutics have been clinically approved to date.

Another way to minimise the systemic toxicity of a chemotherapeutic is to increase its cancer-targeting capability using a wide range of delivery vectors (*e.g.* liposomes, nanotubes, peptides, hormones, and proteins).^1^ A few Pt–protein conjugates have been reported so far. These include albumin adducts bound to octahedral Pt(iv) *via* non-covalent interactions with fatty acid-like axial ligands,^2^ or *via* a maleimide group targeting the free cysteine residue (Cys34) of albumin.^3,4^ A few Pt–antibody conjugates have also been developed, mainly involving Pt(ii) agents, but these have not displayed potential for clinical translation.^5–8^ Development of Pt–protein adducts presents several challenges, including the need for mild, water–compatible conjugation procedures that do not excessively disrupt the protein structure. Release of the cytotoxic payload from the protein vector once the tumour target has been reached is also important to ensure efficacy.

Unlike traditional Pt(iv) prodrugs, photoactivatable Pt(iv) azido complexes are inert and non-toxic in the presence of bio-reductants, but release cytotoxic Pt(ii) species upon irradiation with visible light, providing spatial and temporal control of their chemotherapeutic activity.^9^ Derivatisation of these complexes can provide, for example, cancer-targeting peptide conjugates.^10,11^ A protein adduct has also been synthesised, which exploits a pendant biotin moiety to attach the Pt(iv) agent to avidin, utilising the high biotin–avidin affinity.^12^

In this work, the novel photoactivatable Pt(iv) derivative *trans*,*trans*-[Pt(N_3_)_2_(Py)_2_(OH)(OCO-(PEG)_2_-NHCSNH-Ph-NCS)] (**Pt4**, Fig. 1) has been synthesised with a pendant isothiocyanate group, as a platform for facile, universal attachment to proteins *via* lysine side chain conjugation.

**Fig. 1:**
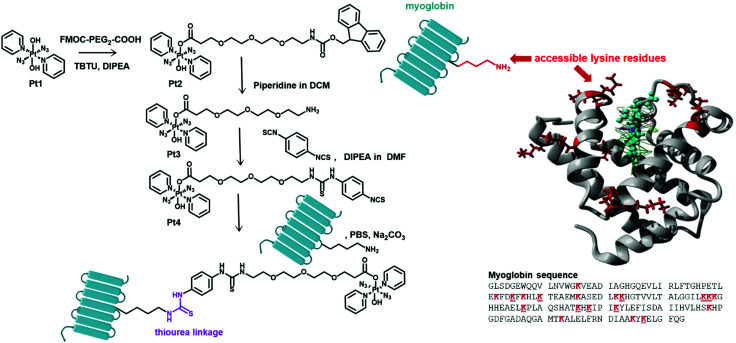
Synthetic route to **Pt4** and its myoglobin derivative **myo-Pt4** (left). Structure and sequence of myoglobin highlighting accessible lysine residues (underlined in structure) based on the X-ray crystal structure of equine myoglobin (right, PDB accession code 1WLA).

**Pt4** was synthesised in 3 steps from the prototype complex **Pt1**. First a Fmoc-protected PEG linker was introduced to enhance biocompatibility and water-solubility of the agent and to act as a spacer between Pt and amino acid residues with the aim of minimising unwanted Pt/protein interactions. Then, upon deprotection of the primary amine function, the resulting intermediate **Pt3** was coupled using a large excess of phenyl bisisothiocyanate to yield a 1 : 1 Pt-isothiocyanate derivative **Pt4**. The identity and the purity of **Pt4** were confirmed by NMR, reversed-phase HPLC, and HR-tandem mass spectrometry (see ESI†). Both **Pt4** and its precursors displayed very high dark stability, but were photoactivated by irradiation with blue light (420 nm), as was evident from the reduction in intensity of the Pt ← N_3_ LMCT band in the UV-vis spectrum (Fig. S1–S3, ESI†).

Myoglobin was used as a model protein to evaluate the ease of protein conjugation of **Pt4** and investigate the properties of the resulting conjugate. Myoglobin (equine, *M*_W_ 17.6 kDa, 153 amino acids, Fig. 1) is a small protein carrying a non-covalently bound haem group, and 10 of its 19 Lys residues are solvent-accessible (based on the crystal structure).^13^ Furthermore, its Soret absorption band (*ca*. 408 nm) is well separated from the LMCT band of the Pt(iv) azido complex (*ca*. 288 nm), enabling precise quantification of protein concentration within the bioconjugate solution.

Myoglobin was reacted with 5 mol equiv. **Pt4** in water at pH 8.5–9 for 4 h followed by gel filtration to remove unreacted **Pt4**. HPLC analysis of the mixture was performed to verify successful conjugation and absence of unreacted **Pt4** using a size-exclusion (4–400 kDa) column and a diode-array detector (DAD). The HPLC chromatograms provided a straightforward differentiation between the unmodified protein and the Pt(iv) conjugate **myo-Pt4**, owing to their different UV-vis absorption spectra (Fig. 2A). Unmodified myoglobin, for which *A*_360_ > *A*_254_ and Δ*A* = *A*_254_ − *A*_360_ < 0, gave a sharp negative HPLC peak at 3 min 45 s. In contrast, **myo-Pt4** displayed one peak at a similar retention time to myoglobin (4 min 4 s) but positive Δ*A*, suggesting that the new chemical entity was similar in size to myoglobin, but had a different UV-vis spectrum. Notably, a broad positive peak at 6 min 33 s was recorded for **Pt4** (Fig. S5, ESI†). DAD spectra for each HPLC peak (Fig. 2A and Fig. S5, ESI† insets), show that **myo-Pt4** displays the peaks corresponding to both the myoglobin haem group (408 nm) and **Pt4** (254 nm, 288 nm). An average **Pt4**/myoglobin ratio of 5.6 was determined for **myo-Pt4** using UV-vis spectroscopy to measure protein concentration from the absorbance of the Soret band ([myoglobin] = 2.9 μM) and ICP-MS to determine **Pt4** concentration ([Pt] = 16.4 μM).

**Fig. 2:**
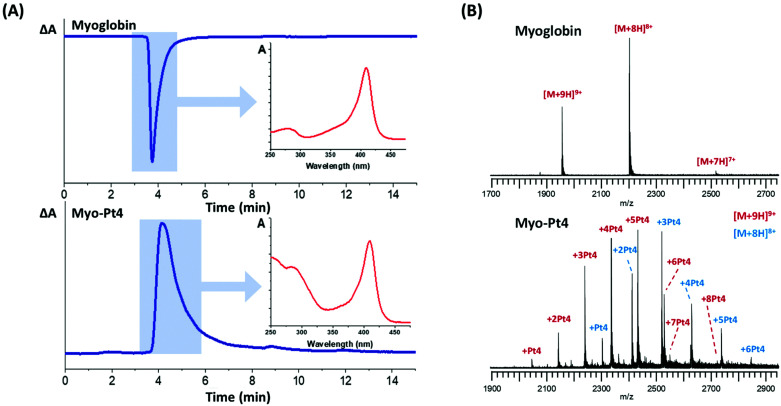
Characterisation of **myo-Pt4** using (A) HPLC (detection *λ*: 254 nm, reference: 360 nm, Δ*A* = *A*_254_ − *A*_360_), insets show DAD spectrum for the peak, and (B) FTICR-MS comparing native *holo*-myoglobin (top panel) with the bioconjugate (bottom panel).

ESI-MS analysis of the mixture identified different myoglobin-**Pt4** conjugates containing up to 8 **Pt4** complexes per myoglobin molecule, while no unmodified myoglobin remained (Fig. 2B). Importantly, no *apo*-myoglobin was observed, demonstrating retention of the haem group throughout the conjugation/purification process and the MS analysis. This is particularly significant as it highlights the gentle reaction and MS conditions employed. Analysis of the relative intensity of the peaks for the most prominent charge state (9+) also allowed calculation of an approximate average Pt/protein ratio of 4.4 **Pt4**/myoglobin in good agreement with the value obtained by UV-vis/ICP-MS measurements (5.6).

**Myo-Pt4** was stable in the dark with no changes in UV-vis absorbance over 2 h, showing that the Pt(iv) azido complex retains its stability when conjugated to myoglobin (Fig. 3).

**Fig. 3:**
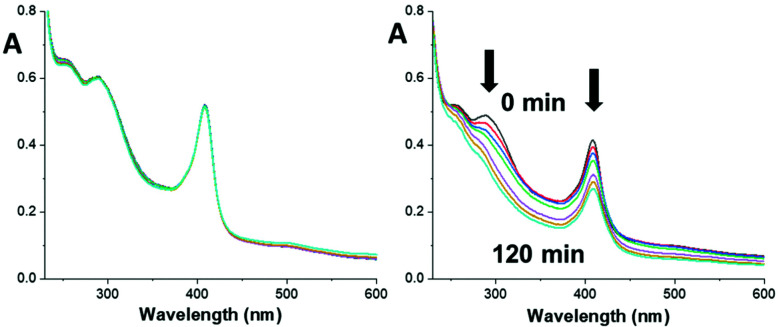
UV-vis spectra of **myo-Pt4** over 2 h in the dark (left) or upon 420 nm light irradiation (right), showing absorbance reduction at 288 nm and 408 nm (myoglobin Soret band).

In contrast, upon irradiation with blue light both the 288 nm peak (Pt ← N_3_ LMCT band) and the 408 nm peak (myoglobin Soret band) decreased significantly indicating photodecomposition (Fig. 3). Interestingly, no photodecomposition was observed when native myoglobin underwent the same treatment (Fig. S6, ESI†) suggesting that the haem group may interact with **Pt4** during photodecomposition. Further investigation is required to confirm this possibility.

Notably, photoactivation was also observed upon green light irradiation (520 nm, 15% decrease in *A*_288_ peak at 2 h Fig. S7, ESI†), although to a lesser extent compared to blue light (30% decrease). Size-exclusion HPLC of the irradiated mixture revealed the presence of several peaks (Fig. S8, ESI†), but MS analysis of the sample was complicated by the complex nature of the mixture. This was unsurprising since a high ratio of Pt/protein, although advantageous to guarantee sufficient cytotoxic payload delivery, complicates the characterisation of the conjugate. Therefore, to gain more insight into the nature of the **myo-Pt4** adducts and the photochemical reactions that take place upon photoactivation, the conjugation was repeated using only 1 mol equiv. of **Pt4**, with the aim of selectively forming a 1 : 1 adduct. MS analysis revealed the presence of unmodified myoglobin in the sample as well as the 1 : 1 conjugate (Fig. S9A, ESI†). Notably, tandem MS analysis of the 1 : 1 conjugate with ECD produced unmodified fragments spanning the entire myoglobin sequence (Table S1, ESI†), suggesting that multiple accessible lysines are susceptible to modification by compound **Pt4**, consistent with myoglobin's X-ray structure (Fig. 1). MS investigation of this new sample after 2 h irradiation with blue light (Fig. S9B, ESI†) showed no intact conjugate, but two main peaks were identified with masses of 17 706.096 Da attributable to loss of [2× N_3_˙ and OH] (calc. mass = 17 706.114 Da) and 17 353.073 Da to loss of [2×(C_5_H_5_N), 2×(N_3_), OH and Pt] (calc. mass = 17 353.065 Da). The first peak showed typical photodecomposition patterns observed previously for the photoactivation of compound **Pt1**, with loss of the azido ligands and an OH group. In contrast, the second peak demonstrates how the platinum complex is at least partially released from the protein upon light activation. Importantly, the complex released upon irradiation is the prototype complex **Pt1** that can then undergo its own photodecomposition pathways, as previously investigated,^14^ leaving the PEG isothiocyanate linker bound to the protein.

Encouraged by these results for **myo-Pt4**, we investigated **Pt4** conjugation to antibodies, which are sensitive to the solution environment and can easily precipitate, form aggregates, or denature when manipulated at pH and concentrations of salts that differ from physiological conditions.

As a proof of concept, we investigated conjugation to the Her2-targeting monoclonal antibody trastuzumab (Herceptin®, *M*_W_ ≈ 148 kDa) used in the treatment of Her2-positive breast and gastric cancers.^15^ In addition to being clinically relevant, trastuzumab can form stable antibody–drug-conjugates (ADCs) with chemotherapeutics *via* its lysine residues, as shown in the clinically approved ADC with the microtubule inhibitor maytansine (trastuzumab emtansine).^16^

Trastuzumab was reacted with 5 mol equiv. **Pt4** in PBS at pH ≈ 8.5 for 2 h, followed by purification using ultracentrifugation. As expected, ESI-MS analysis of **trastuzumab-Pt4** (Fig. 4) revealed a more complex pattern of modification compared to **myo-Pt4** owing to the higher *M*_W_, as well as heterogeneity of trastuzumab in terms of glycosylation and other post-translational modifications.^17^ Notably, while peaks for unmodified trastuzumab were still visible in the mixture, the presence of **Pt4**:trastuzumab adducts was evident. Due to the aforementioned heterogeneity, the peaks for the unmodified antibody possess an intrinsic width of around 900 Da,^17^*i.e.*, on the order of the mass of **Pt4**. As a result, it is fundamentally impossible to baseline-separate the different adducts as for myoglobin. Specifically, the most heavily glycosylated portion of the Pt-free population overlaps with the least glycosylated portion of the 1 : 1 Pt : antibody adduct, and similarly for other adducts (see Fig. 4). To overcome the limitations due to the broad trastuzumab peaks, we simulated spectra of trastuzumab with zero, one, and two additions of **Pt4** (**Apo**, **1 Pt4**, **2 Pt4** in Fig. 4, respectively). A linear combination (blue dashed line) of these simulated spectra was then able to approximate satisfactorily the experimentally observed shape of the signal for the 48+ charge state of the modified protein (solid black line). This analysis suggests that *ca.* 55% of the trastuzumab present was unmodified, while the adducts with 1 and 2 **Pt4** per antibody represent 30 and 15% of the intensity, respectively, resulting in an average **Pt4**/trastuzumab ratio of *ca.* 0.6. This is in good agreement with the ratio obtained for the same batch by ICP-MS/UV-vis measurements (Pt/trastuzumab = 0.7). This lower degree of labelling for trastuzumab, compared to myoglobin, can be partially explained by considering the milder conjugation conditions employed in this case to avoid antibody denaturation (shorter conjugation time, lower pH). In particular, isothiocyanate coupling is known to be very sensitive to pH. While a lower **Pt4**/antibody ratio can be beneficial in this context, as it reduces heterogeneity, simplifying the immunoconjugate characterisation, higher **Pt4**/antibody ratios could be used to increase **Pt4** delivery to cancer cells to maximise the biological effect. Immunoconjugates with higher **Pt4**/antibody ratio should be obtainable by increasing the molar equivalents of **Pt4** used in the conjugation reaction as previously described.^18^

**Fig. 4:**
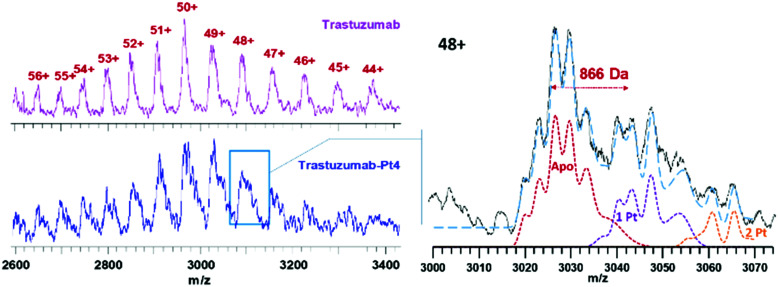
Charge state distribution of trastuzumab before (top left) and after (bottom left) conjugation with **Pt4**. The panel on the right zooms in on the 48+ peak, showing the simulated spectra for trastuzumab with zero (**Apo**, red line), one **Pt4** (purple line), and two **Pt4** (orange line). Linear combination (blue line) of the three simulated spectra with relative intensities of 55 : 30 : 15 is in agreement with the experimental data.

Overall, this work has shown that the new Pt(iv) isothiocyanate derivative **Pt4** undergoes facile conjugation to proteins and light-mediated release of its photoactivatable cytotoxic payload: prototype complex **Pt1**. Thus, **Pt4** is a useful platform for protein-mediated delivery of photoactivatable Pt(iv) agents. Our proof-of-concept conjugation experiment shows that **Pt4** can conjugate to monoclonal antibodies under mild conditions, producing, for the first time, an antibody–drug-conjugate containing a photoactivatable Pt(iv) agent.

This research was funded by the Wellcome Trust [209173/Z/17/Z] and EPSRC [EP/P030572/1]. For the purpose of open access, the author has applied a CC BY public copyright licence to any Author Accepted Manuscript version arising from this submission. F. L. acknowledges funding by the LOEWE project TRABITA funded by the Ministry of Higher Education, Research and the Arts (HMWK) of the State of Hesse.

## Conflicts of interest

There are no conflicts to declare.

## Supplementary Material

CC-057-D1CC02722K-s001
