# Supplementary material for: Facile protein conjugation of platinum for light-activated cytotoxic payload release
Source: Chem Commun (Camb). 2021 Jul 12;57(62):7645–8. doi: 10.1039/d1cc02722k (PMC8330822; doi:10.1039/d1cc02722k)
Supplement: CC-057-D1CC02722K-s001 [file CC-057-D1CC02722K-s001.pdf]

Electronic Supplementary Material (ESI) for Chemical Communications.
This journal is © The Royal Society of Chemistry 2021

Supporting Information

Facile protein conjugation of platinum for light-activated cytotoxic payload release

Cinzia Imberti, Frederik Lermyte, Emily P. Friar, Peter B. O'Connor and Peter J. Sadler

Contents

Material and Methods

Synthesis and characterisation data

Conjugation to myoglobin

Size exclusion HPLC

Conjugation to trastuzumab

Determination of platinum/protein ratios

Dark stability and photodecomposition

FT-ICR Mass spectrometry

Table S1 Tandem MS-MS analysis of **myo-Pt4 1:1**

Figure S1-S3 Dark stability and photoactivation of **Pt4** and intermediates

Figure S4 Tandem MS of **Pt4**

Figure S5 Size-exclusion HPLC of **Pt4**

Figure S6 Light stability of myoglobin

Figure S7 UV-vis spectra of **myo-Pt4** upon green light irradiation and % absorbance loss

Figure S8 Size-exclusion HPLC of irradiated **myo-Pt4**

Figure S9 FT-ICR analysis of **myo-Pt4 1:1**

Figures S10-12 HPLC characterisation of **Pt4** and intermediate complexes

Figures S13-17 NMR spectra of **Pt4** and intermediate complexes

Materials and Methods

TBTU and formic acid were obtained from Fluka Chemicals and trifluoroacetic acid (TFA) from Fisher Scientific. trans,trans,trans-[Pt(N_3)$_2$(Py)$_2$(OH)$_2$] (**Pt1**, Py = pyridine) was synthesised according to published procedures.[1] Trastuzumab (Herceptin®, Roche, 24.12 mg/mL in saline) was obtained from the Pharmacy Department at Guy's and St. Thomas' NHS Trust, London. All other chemicals were obtained from Sigma Aldrich and used without further purification. All H_2O used in reactions was MilliQ ultrapure water to minimize unwanted metal contaminants. Silica gel used was technical grade, pore size 60 Å, 230-400 mesh, 40-63 µm particle size.

NMR spectra were recorded on a Bruker Avance III 500 MHz (for ^{1}H) spectrometer and referenced to the residual solvent signal of the solvent. ESI-HRMS spectra in positive mode were recorded on a Bruker microTOF instrument in the range of 200–3000 m/z. Electronic absorption spectra were recorded on a Varian Cary 300 UV-vis spectrophotometer in a quartz cuvette at 298 K using neat solvent as reference. Platinum content was analysed on an ICP-MS 7500cx (Agilent).

LC-MS experiments were carried out on Bruker Amazon X ion trap instrument connected online with an Agilent 1260 HPLC. An Agilent ZORBAX Eclipse XDB-C18 column (250×4.6 mm, 5 µm) was used with 0.7 mL/min flow rate. The mobile phase contained 0.1% v/v formic acid in H_2O (solvent A) and in CH_3CN (solvent B). **Gradient 1** included 5 min of equilibration at 10% B at the start of the run, then concentration of B increased from 10% (5 min) to 80% (at 30 min, maintained until 35 min) and subsequently decreased to 10% (at 40 min).

FTICR-MS experiments were carried out on a 12 T Bruker solariX instrument.

Synthesis and characterisation data

No problems were encountered during this work. However, heavy metal azides are known to be shock sensitive detonators therefore extra care was taken during handling.

All synthesis, purification and analysis, with the exception of the irradiation studies, were carried out in the dark with minimal light exposure.

Trans,trans,trans-[Pt(N_3)$_2$(Py)$_2$(OH)(OCO-(PEG)$_2$-NH-Fmoc)] (Pt2)

DIPEA (29.5 µL, 21.9 mg, 169.6 µmol) was added to a solution of TBTU (34.0 mg, 106 µmol) and Fmoc-NH-(PEG)$_2$COOH (47.1 mg, 106 µmol) in DMF (1.2 mL). This was subsequently added to **Pt1** (50 mg, 106 µmol) in DMF (1.2 mL) and stirred at 45 °C for 24 h. The crude

product was then purified via column silica gel chromatography with increasing percentage of MeOH in DCM (0% - 5%). Purification was monitored used TLC using 10% DCM in MeOH as a mobile phase. Rf = 0.68. 26 mg of pure product were isolated (27% yield).

This complex was observed by DLS to form aggregates when in 5% DMSO in water, resulting in its UV-vis absorbance to increase over time as the aggregate dissolved (Figure S1). Similarly, 2 species were observed for this complex by NMR with a 80:20 ratio, identical coupling pattern, but slightly different chemical shift suggesting they belong to the same complex in different chemical environment. NMR peak assignments below are reported for the major species only.

^{1}H NMR (500 MHz, CD_3OD) δH = 8.90 (dd, J = 5.5 Hz, J^{195}Pt–^{1}H = 26.5 Hz, 4H, H_o in pyridine), 8.19 (t, J = 7.5 Hz, 2 H, H_p in pyridine), 7.80 (d, J = 7.5 Hz, 2 H in FMOC) 7.74 (t, J = 6.5 Hz, 4 H, H_m py), 7.65 (d, J = 7.5 Hz, 2 H in FMOC), 7.39 (t, J = 7.5 Hz, 2 H, in FMOC), 7.31 (d, J = 7.5 Hz, 2 H in FMOC), 4.37 (d, J = 6.5 Hz, 2 H, COOC**H**$_2$CH in FMOC) 4.20 (t, J = 6.5 Hz, 1 H, COOCH$_2$C**H** in FMOC) 3.69 (2 H, t, J = 6 Hz, Pt-OCOCH$_2$**CH$_2$**) 3.57-3.63 (m, 8 H, OC**H**$_2$CH$_2$O and OCH$_2$C**H**$_2$O in PEG$_2$), 3.50 (t, J = 5.4, 2H, OC**H**$_2$CH$_2$NH-FMOC), 3.26 (t, J = 5.4, 2H, OCH$_2$C**H**$_2$NH-FMOC), 2.57 (t, J = 6 Hz, 2 H, Pt-OCOC**H**$_2$CH$_2$). 5.49 (DCM), 1.29, 0.9 (grease).
^{13}C NMR (126 MHz, CD_3OD) δC = 177.8 (O**C**OCH2), 150.9 (Co, pyridine), 144.0 (O**C**ONH), 143.1 (Cp, pyridine), 142.6 (aromatic CH in FMOC), 128.8 (Cm, pyridine), 128.8, 128.2, 127.4, 126.2, 121.2 (other aromatic CH in FMOC), 71.6, 71.3, 71.2, 70.9, 69.3 (**C**H$_2$O in PEG), 67.2 (NHCOO**C**H2CH in FMOC), 55.9 (NHCOOCH2**C**H in FMOC), 43.8 (OCH$_2$**C**H$_2$NH-FMOC), 41.8 (Pt-OCO**C**H$_2$), 38.6, 18.7 17.3 (DIPEA contamination).
HRMS: m/z $[M+H]^+$ calcd. 897.2644 found 897.2632, $[M+Na]^+$ calcd. 919.2464 found 919.2452.

***Trans,trans,trans*-[Pt(N$_3$)$_2$(Py)$_2$(OH)(OCO-(PEG)$_2$-NH$_2$)] (Pt3)**
Piperidine (10 µL, 9.2 mg, 107 µmol) in DCM (1 mL) was added to **Pt2** (24. 1 mg, 27 µmol) and stirred at room temperature for 3 hrs. The crude mixture was diluted with DCM (4 mL), extracted 4x with water (5 mL). Water and piperidine were removed by rotary evaporation and the crude product redissolved in the minimum amount of water and purified via a C18 cartridge (SEP-PAK Plus Short, Waters) with increasing percentage of MeCN in H_2O (0% - 30%).

^{1}H NMR (500 MHz, D_2O) δ_H = 8.80 (dd, J = 6 Hz, J^{195}Pt–^{1}H = 27 Hz, 4 H, H_o) 8.28 (2H, t, J = 7 Hz, H_p) 7.81 (4H, t, J = 7 Hz, H_m) 3.70 (2 H, t, J = 6 Hz, Pt-OCOCH$_2$**CH$_2$**) 3.64 (4 H, broad

s, 1 PEG unit OC**H**$_2$C**H**$_2$O) 3.61 (2 H, t, J = 5 Hz, O**CH$_2$**CH$_2$NH$_2$) 3.58 (4 H, broad s, 1 PEG unit OC**H**$_2$C**H**$_2$O) 2.93 (2 H, t, J = 5 Hz, OCH$_2$**CH$_2$**NH$_2$) 2.64 (2 H, t, J = 6 Hz, Pt-OCO**CH$_2$**CH$_2$O).

^{13}C NMR (126 MHz, D$_2$O) δ_C = 178.1 (CO) 149.2 (Co, pyridine) 142.7 (Cp, pyridine) 127.0 (Cm, pyridine) 69.6 (Pt-OCOCH$_2$**C**H$_2$O) 69.4, 69.3 (O**C**H$_2$CH$_2$O), (OCH$_2$**C**H$_2$O) 67.5 (O**C**H$_2$CH$_2$NH$_2$), 39.5 (OCH$_2$**C**H$_2$NH$_2$), 36.8 (Pt-OCO**CH$_2$**CH$_2$O).

HRMS: m/z $[M+H]^+$ calcd. 675.1962 found 675.1965.

***Trans,trans,trans*-[Pt(N$_3$)$_2$(Py)$_2$(OH)(OCO-(PEG)$_2$-NH-CSNH-Ph-NCS)] (Pt4)**

Complex 3 (5 mg, 7.4 mmol) was dissolved in 1 mL DMF and 5 µL of DIPEA (29 mmol) was added. Para-phenylene diisothiocyanate (14 mg, 280 mmol) was separately dissolved in 500 µL DMF and added to the mixture that was stirred at room temperature for 40 min. DMF was removed by rotary evaporation at room temperature and the brownish residue dissolved in DCM. The product was purified by column chromatography (0-6% methanol in DCM). Purification was monitored by TLC using 10% DCM in MeOH as a mobile phase.

^{1}H NMR (500 MHz, acetone-d6) δ_H = 8.99 (dd, J = 6 Hz, J^{195}Pt–^{1}H = 27 Hz, 4 H, H$_o$) 8.27 (2H, t, J = 7.2 Hz, H$_p$) 7.82 (4H, t, J = 7.2 Hz, H$_m$), 7.65 (t, J = 7.8 Hz, 2 H, NHCSNHC-C**H**), 7.28 (d, J = 9 Hz, 2 H, NHCSNHC-CH-C**H**), 3.72 (2 H, t, J = 6 Hz, Pt-OCOCH$_2$C**H$_2$**) 3.67 (2 H, m, OC**H$_2$**CH$_2$NH$_2$) 3.64-3.59 (10 H, OC**H**$_2$CH$_2$O and OCH$_2$C**H**$_2$O in PEG$_2$) 2.56 (2 H, t, J = 6 Hz, Pt-OCOC**H**$_2$), 5.62 (DCM signal), 3.31, 3.30 (MeOH), 2.82, 2.78 (H$_2$O), 2.05 (acetone), 1.29, 0.9 (grease).

HRMS: m/z $[M+H]^+$ calcd. 866.17563 found 866.17562

Conjugation to myoglobin

700 µL of a 17 µM myoglobin solution in water was treated with a 1 M sodium carbonate solution to reach pH 8.5-9 (pH paper). A 1.7 mM solution of **Pt4** in DMSO was then added in 5 µL aliquots to the myoglobin solution in a microcentrifuge tube, vortexing the tube after each addition to ensure full mixing. For the 5 mol. equiv. batch, 34 µL of **Pt4** was added in total; for the 1 mol. equiv. batch only 7.5 µL was added. The reaction mixture was then vigorously mixed and left to incubate at room temperature for 4 h, followed by purification by size-exclusion chromatography using a PD10 column (GE Healthcare) preconditioned with 25 mM ammonium acetate.

Size-exclusion HPLC

Size-exclusion HPLC analyses were carried out on an Agilent 1100 HPLC equipped with a DAD detector. An Agilent ZORBAX GF250 4-400 kDa size-exclusion column (250×4.6 mm, 4 μm) was utilised for the analysis, using 50 mM ammonium acetate in water as a mobile phase and a 1 mL/min flow rate. Detecting wavelength was set at 254 nm, reference wavelength at 360 nm.

Conjugation to trastuzumab

A PBS solution of trastuzumab (730 μL, 16.6 μM) was treated with 1 M sodium bicarbonate to reach pH 8.5, then 34 μL of a 1.7 mM DMSO solution of **Pt4** (5 mol. equiv.) was added in small aliquots and vortexing the solution between additions to ensure good mixing. After the final addition, the reaction mixture was vortexed and then left to incubate at room temperature for 2 h, followed by purification and buffer exchange to 100 mM ammonium acetate using 8 cycles of ultracentrifugation (Vivaspin MWCO 50,000).

Determination of platinum/protein ratios

Protein concentration for **myo-Pt4** samples was determined by UV-vis spectroscopy using (ε_{408} = 179,000 mol L^{-1} cm^{-1}).[2] Trastuzumab concentration in **Trastuzumab-Pt4** samples was determined using a NanoDrop Lite spectrophotometer (Thermo Scientific) using settings for IgG. The concentration of platinum in samples of both **myo-Pt4** and **Trastuzumab-Pt4** was determined using ICP-MS. Samples were prepared in a 3.6% v/v HNO_3 solution and analysed using a no-gas mode.

Dark stability and photodecomposition

Complexes **Pt4** and its precursors were dissolved in 5% DMSO 95% water v/v; **myo-Pt4** was in 25 mM ammonium acetate. For dark stability, the UV-Vis spectrum was monitored over time while keeping the solution in the dark. For photodecomposition studies, the UV-Vis spectrum was measured at the same time point following irradiation. For blue light irradiation (λ = 420 nm), a LZC-ICH2 photoreactor (Luzchem Research Inc.) was used equipped with a temperature controller and 8 Luzchem LZC-420 lamps without light filtration. For green light irradiation (λ = 517 nm), an LED source was used (BASETech model no. SP-GU10 230 V~50 Hz 1.3-2.1 W).

FT-ICR Mass spectrometry

A 12 T solariX instrument (Bruker Daltonik GmbH, Bremen, Germany) equipped with an infinity cell was used for these experiments. Nano-electrospray ionisation was performed in positive-ion mode using a home-built source. To produce emitters, 1.2-mm thin-walled glass capillaries (World Precision Instruments, Hitching, UK) were pulled in-house with a P97 Flaming/ Brown type micropipette puller (Sutter Instrument Co., Novato, CA, USA) to obtain tips of ca. 1-µm orifice diameter. Data analysis was performed using Bruker Compass DataAnalysis 4.1, and peaks were assigned manually. For native MS, myoglobin was dissolved at a concentration of 10 µM in 25 mM aqueous ammonium acetate. MS measurements of myoglobin and trastuzumab under denaturing conditions were performed using 40 – 50% acetonitrile and 0.1 – 0.5% formic acid.

Table S1. ECD of [[Mb-**Pt4**] + 15H]$^{15+}$. Fragment assignment in ECD of [Mb + 15H]$^{15+}$ (first part) and [Mb-**Pt4** + 15H]$^{15+}$ (second part).

**Fragment**	**m/z (theory)**	**m/z (observed)**	**Error (ppm)**
[c5]+	447.2198	447.2199	0.2
[c17]2+	957.4971	957.4973	0.2
[c30]2+	1623.8308	1623.8308	0.0
[c31]2+	1701.8813	1701.8815	0.1
[c34]2+	1882.4814	1882.4803	-0.6
[c31]3+	1134.9233	1134.9235	0.1
[c35]3+	1274.3305	1274.3322	1.3
[c41]3+	1509.7734	1509.7740	0.4
[c31]4+	851.4443	851.4451	0.9
[c40]4+	1100.3212	1100.3223	0.9
[c59]4+	1669.6078	1669.6079	0.1
[c51]5+	1160.8138	1160.8145	0.6
[c59]5+	1335.8877	1335.8876	-0.1
[c65]5+	1471.5640	1471.5648	0.6
[c66]5+	1491.7735	1491.7717	-1.2
[c59]6+	1113.4076	1113.4080	0.3
[c67]6+	1259.8239	1259.8237	-0.1
[c67]7+	1079.9929	1079.9938	0.8
[c69]7+	1110.3004	1110.3005	0.1
[c77]7+	1217.9397	1217.9399	0.1
[c92]7+	1454.2104	1454.2110	0.4
[c98]7+	1554.5522	1554.5516	-0.4
[c69]8+	971.6388	971.6399	1.1
[c78]8+	1081.8350	1081.8353	0.2
[c92]8+	1272.5600	1272.5609	0.7
[c93]8+	1289.6924	1289.6911	-1.0
[c97]8+	1344.3472	1344.3469	-0.2
[c98]8+	1360.3591	1360.3593	0.1
[c91]9+	1121.6062	1121.6066	0.4
[c93]9+	1146.5052	1146.5064	1.1
[c97]9+	1195.0872	1195.0878	0.5
[c98]9+	1209.3200	1209.3198	-0.1
[c115]9+	1427.6682	1427.6696	1.0
[c98]10+	1088.4887	1088.4895	0.7
[z7]+	762.3913	762.3913	0.0
[z8]+	925.4546	925.4544	-0.2
[z10]2+	562.7970	562.7968	-0.4
[z17]2+	977.5193	977.5202	0.9
[z18]2+	1042.0406	1042.0404	-0.2
[z24]3+	900.1433	900.1441	0.8
[z25]3+	919.1505	919.1514	1.1
[z28]3+	1023.8580	1023.8585	0.5

[z32]3+	1153.9093	1153.9097	0.3
[z37]4+	992.2488	992.2489	0.0
[z38]4+	1026.5136	1026.5141	0.5
[z55]4+	1517.7971	1517.7967	-0.2
[z56]4+	1549.8208	1549.8212	0.2
[z44]5+	950.6956	950.6960	0.3
[z55]5+	1214.4391	1214.4393	0.1
[z56]5+	1240.0581	1240.0585	0.3
[z60]5+	1327.5058	1327.5050	-0.6
[z62]5+	1372.3240	1372.3239	-0.1
[z55]6+	1012.2005	1012.2004	0.0
[z60]6+	1106.4227	1106.4230	0.3
[z62]6+	1143.7712	1143.7714	0.1
[z64]6+	1176.9538	1176.9536	-0.2
[z68]6+	1252.1732	1252.1740	0.7
[z84]6+	1530.4942	1530.4956	0.9
[z67]7+	1057.2803	1057.2808	0.5
[z68]7+	1073.4352	1073.4354	0.2
[z70]7+	1102.0180	1102.0184	0.4
[z75]7+	1186.0575	1186.0581	0.4
[z80]7+	1263.1118	1263.1118	0.0
[z84]7+	1311.9961	1311.9963	0.1
[z86]7+	1342.3036	1342.3046	0.8
[z94]7+	1467.8032	1467.8041	0.6
[z76]8+	1053.9381	1053.9382	0.1
[z80]8+	1105.3487	1105.3504	1.5
[z84]8+	1148.1225	1148.1226	0.1
[z86]8+	1174.6415	1174.6429	1.2
[z87]8+	1187.0251	1187.0255	0.3
[z94]9+	1141.8486	1141.8489	0.3
[z98]9+	1187.9826	1187.9833	0.6
[z99]9+	1202.5427	1202.5432	0.4
[z122]9+	1505.4768	1505.4765	-0.2
[z94]10+	1027.7644	1027.7655	1.0
[z105]10+	1149.5241	1149.5250	0.8
[z118]10+	1313.2077	1313.2093	1.2
[z122]10+	1355.0298	1355.0307	0.7
[z119]11+	1199.1005	1199.1010	0.4
[z122]11+	1231.9369	1231.9371	0.1

**Fragment**	**m/z (theory)**	**m/z (observed)**	**Error (ppm)**
[c5]+	447.2198	447.2199	0.1
[c17]2+	957.4971	957.4973	0.2
[c30]3+	1082.8896	1082.8899	0.2
[c31]3+	1134.9233	1134.9230	-0.3
[c41]3+	1509.7734	1509.7732	-0.2

[c31]4+	851.4443	851.4450	0.8
[c55]4+	1565.8061	1565.8077	1.0
[c43]5+	961.2996	961.3004	0.8
[c48]5+	1092.3685	1092.3681	-0.4
[c59]5+	1335.8877	1335.8868	-0.7
[c65]5+	1471.5640	1471.5635	-0.3
[c66]5+	1491.7735	1491.7724	-0.8
[c77]6+	1420.7618	1420.7620	0.1
[c93]7+	1289.6924	1289.6910	-1.1
[c97]7+	1344.3472	1344.3483	0.8
[c97]9+	1195.0872	1195.0876	0.3
[c98]9+	1209.3200	1209.3200	0.0
[c98]10+	1088.4887	1088.4894	0.6
[z7]+	762.3913	762.3912	-0.1
[z8]+	925.4546	925.4547	0.1
[z9]2+	527.2784	527.2784	0.0
[z13]2+	712.3711	712.3713	0.4
[z14]2+	769.3925	769.3933	1.0
[z16]2+	920.9773	920.9770	-0.3
[z17]2+	977.5193	977.5198	0.5
[z18]2+	1042.0406	1042.0406	0.0
[z18]3+	695.0295	695.0300	0.8
[z21]3+	799.1015	799.1019	0.4
[z24]3+	900.1433	900.1441	0.9
[z25]3+	919.1505	919.1512	0.8
[z27]3+	985.5157	985.5155	-0.2
[z35]4+	938.4671	938.4672	0.1
[z36]4+	970.4908	970.4912	0.4
[z37]4+	992.2488	992.2495	0.7
[z38]4+	1026.5136	1026.5141	0.6
[z55]4+	1517.7971	1517.7948	-1.5
[z60]4+	1659.1305	1659.1300	-0.3
[z44]5+	950.6956	950.6960	0.4
[z49]5+	1068.9465	1068.9460	-0.4
[z55]5+	1214.4391	1214.4388	-0.3
[z55]6+	1012.2005	1012.2013	0.8
[z56]6+	1033.5496	1033.5500	0.3
[z57]6+	1056.3928	1056.3929	0.1
[z60]6+	1106.4227	1106.4227	0.0
[z75]6+	1383.5659	1383.5645	-1.0
[z68]7+	1073.4352	1073.4357	0.5
[z70]7+	1102.0180	1102.0188	0.7
[z76]8+	1053.9381	1053.9387	0.6
[z94]9+	1141.8486	1141.8489	0.3
[z106]9+	1292.3658	1292.3658	0.0
[z94]10+	1027.7644	1027.7647	0.2
[c151+Pt4]13+	1356.4743	1356.4721	-1.6
[z152+Pt4]13+	1365.0914	1365.0915	0.1

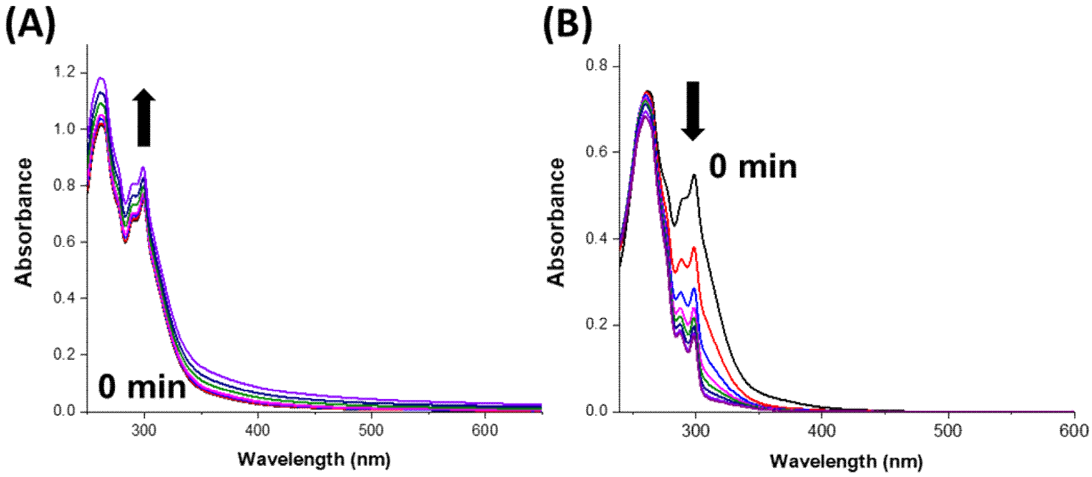

Figure S1. UV-vis spectra of complex **Pt2** (in 5% DMSO /95% water) at different time points over 2 h in the dark (**Pt2** concentration**:** 50 µM, left) or upon irradiation with blue light (**Pt2** concentration**:** 30 µM, right). Decrease of the LMCT attributed to Pt←N_3 is evident upon irradiation. In contrast, an overall increase in absorbance is observed for the complex in the dark, attributed to the dissociation of aggregates over time.

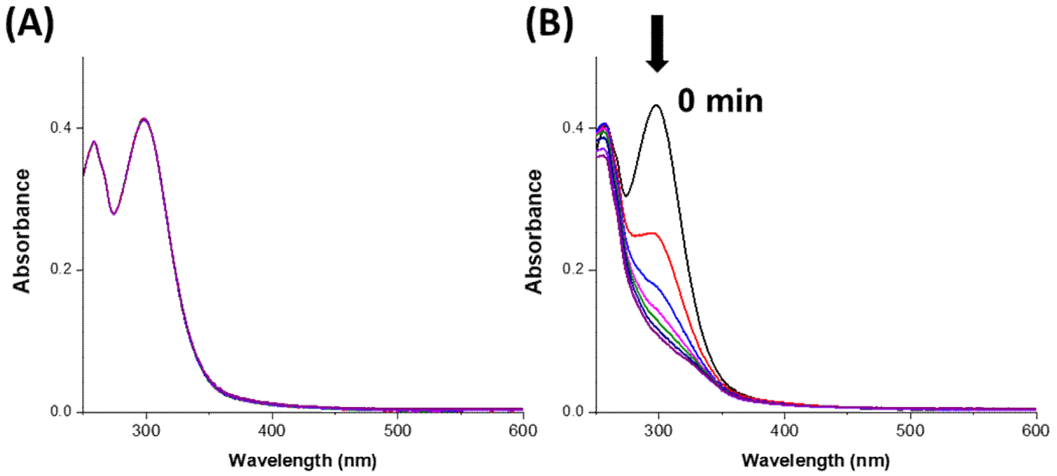

Figure S2. UV-vis spectra of complex **Pt3** (50 µM in 5% DMSO /95% water) at different time points over 2 h in the dark (left) or upon irradiation with blue light (right).

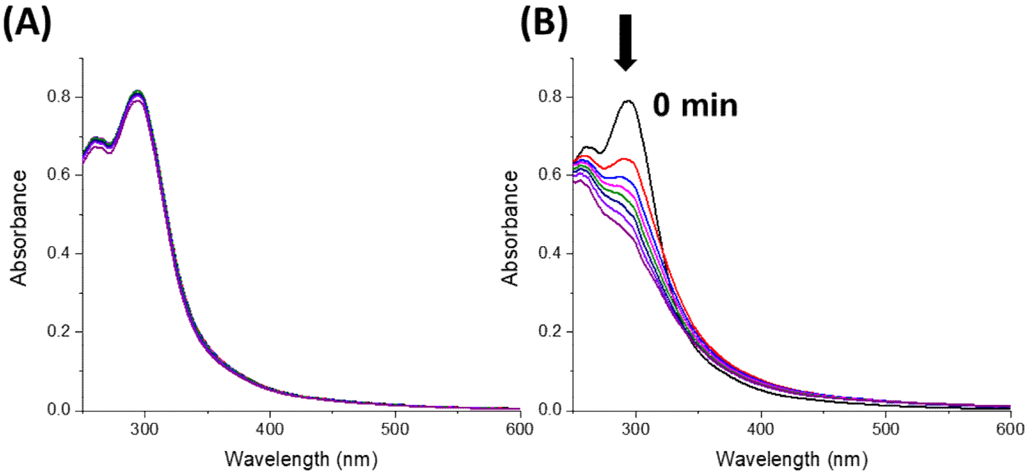

Figure S3. UV-vis spectra of complex **Pt4** (50 µM in 5% DMSO /95% water) at different time points over 2 h in the dark (left) or upon irradiation with blue light (right).

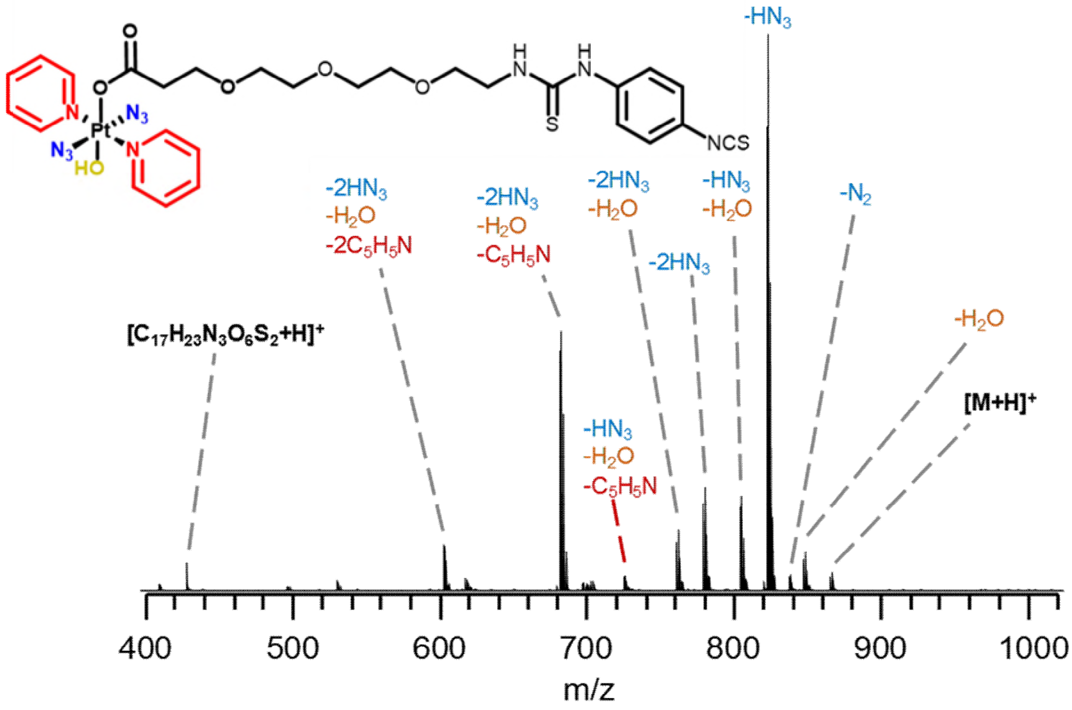

Figure S4. Tandem MS of **Pt4** showing its typical fragmentation pattern.

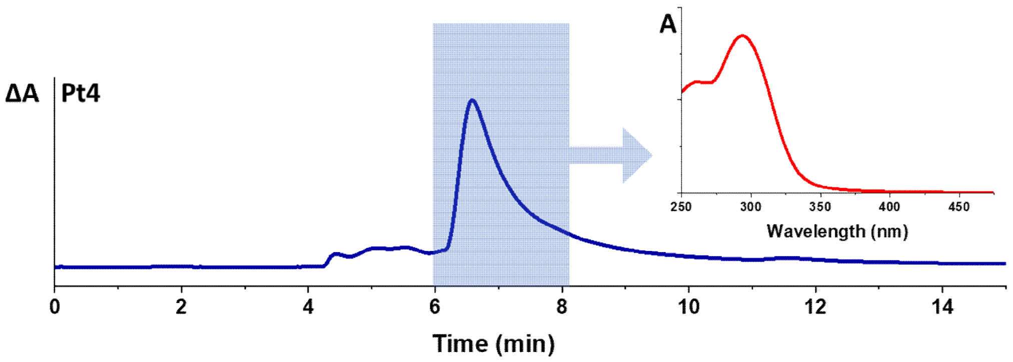

Figure S5. **Pt4** size-exclusion HPLC (detection λ: 254 nm, reference: 360 nm, ΔA= A_{254}-A_{360}), inset shows the DAD spectrum for the main peak and corresponds to the UV-vis spectrum of **Pt4**.

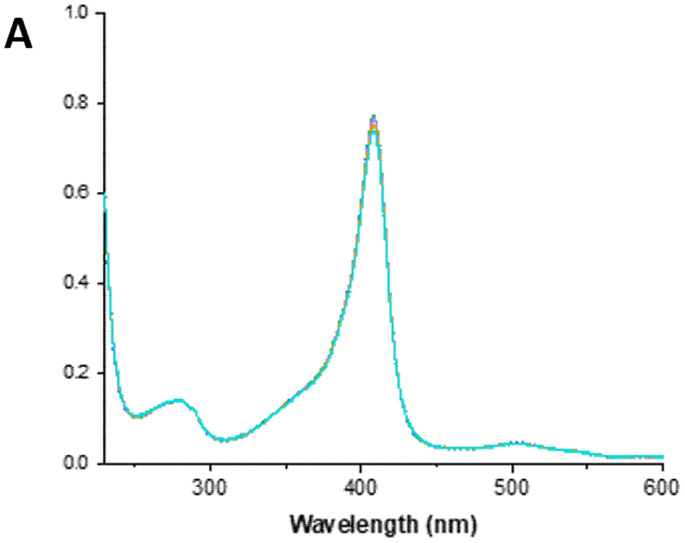

Figure S6. UV-vis spectra of myoglobin (5.7 µM in 25 mM aqueous ammonium acetate) at different time points over 2 h upon irradiation with blue light, showing no photodecomposition.

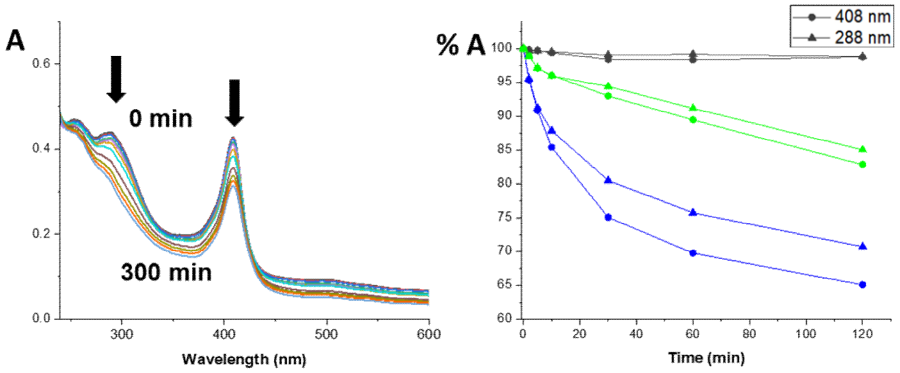

Figure S7. (A) UV-vis spectra of the conjugate **myo-Pt4** (25 mM aqueous ammonium acetate) over 5 h upon green light irradiation (3 µM myoglobin), showing reduction of the absorption band at 288 nm as well as the Soret band at 408 nm. (B) Percentage change in absorbance (288 nm - ● and 408 nm - ▲) at different time points in the dark (black lines), or upon irradiation (blue and green lines indicate 420 nm and 520 nm, respectively).

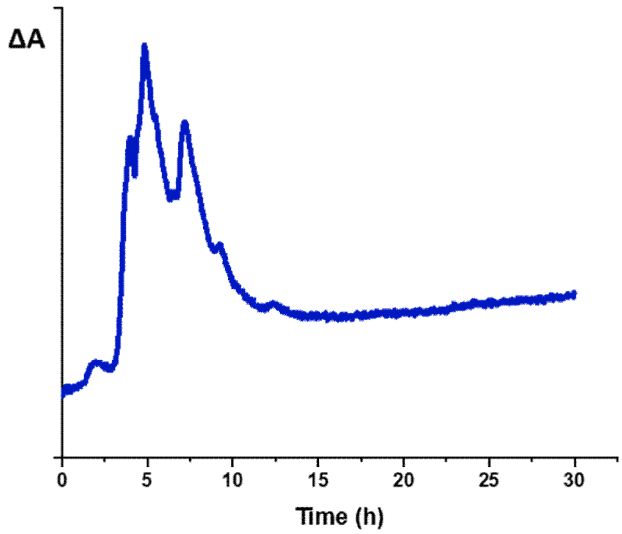

Figure S8. Size-exclusion HPLC of myo-Pt4 after 2h irradiation (detection λ: 254 nm, reference: 360 nm, $\Delta A = A_{254} - A_{360}$). Photodecomposition of the conjugate is evident from the appearance of multiple peaks (compare to Fig. 2), but the mixture is too complicated for MS analysis.

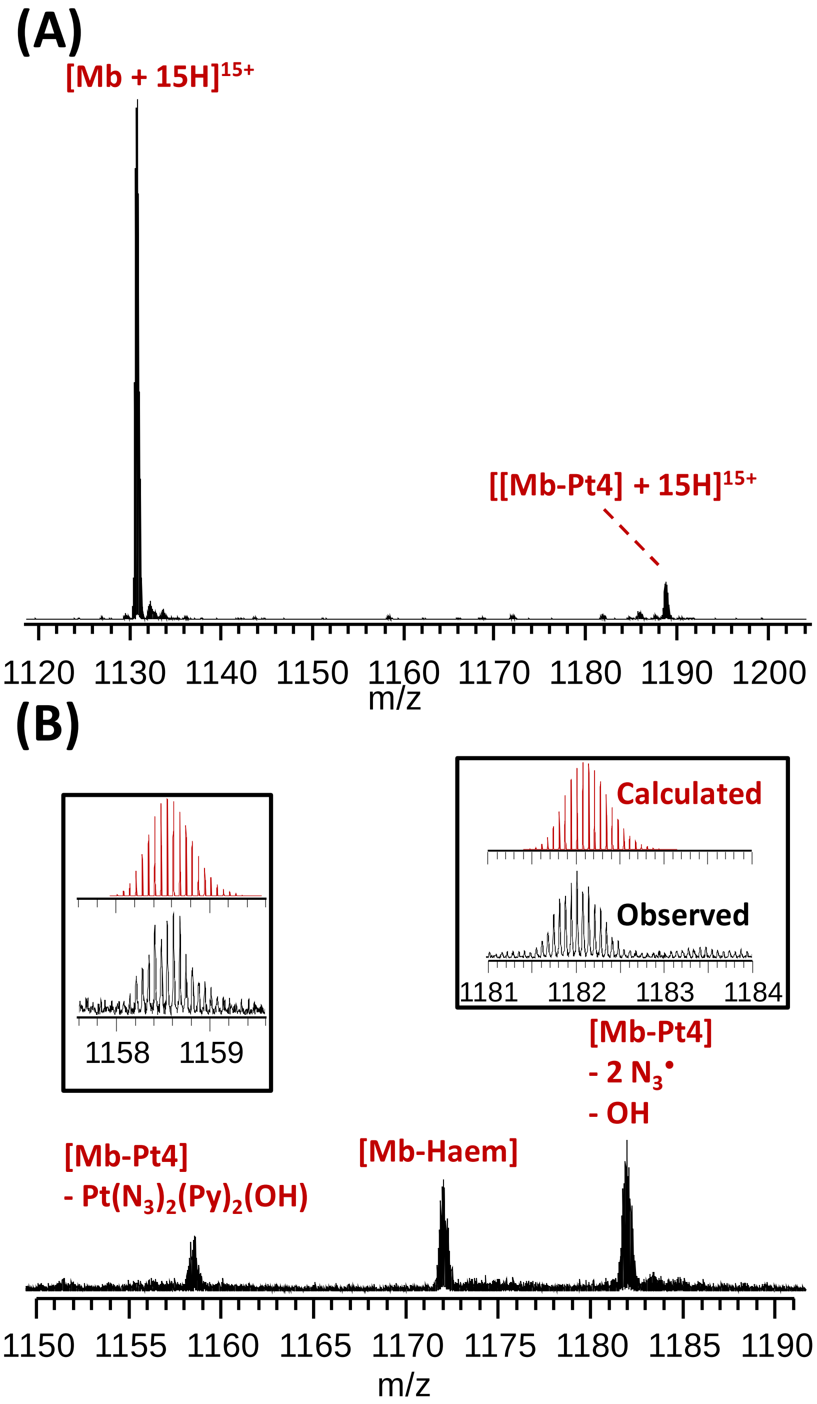

Figure S9. FTICR-MS analysis of a 1:1 myo:Pt4 conjugation batch sample, showing the presence of free protein and 1:1 conjugate (A). Decomposition products of the 1:1 conjugate after 2h irradiation with blue light are shown in (B), with calculated and observed isotope distributions shown in insets. The 15+ charge state is shown as it is the most intense state present in the spectrum.

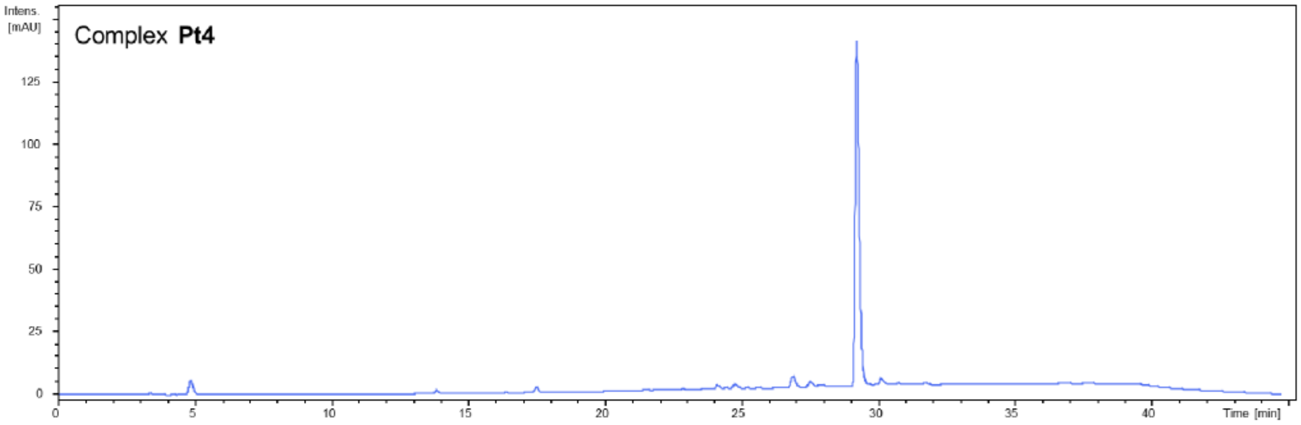

Figure S10. HPLC UV-vis chromatogram of **Pt4** (detection wavelength 254 nm), gradient 1.

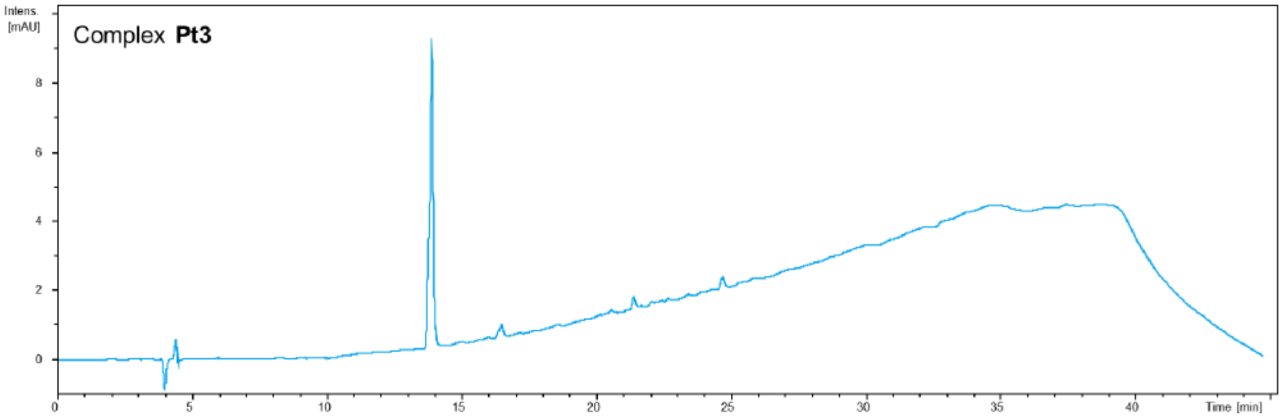

Figure S11. HPLC UV-vis chromatogram of **Pt3** (detection wavelength 254 nm), gradient 1.

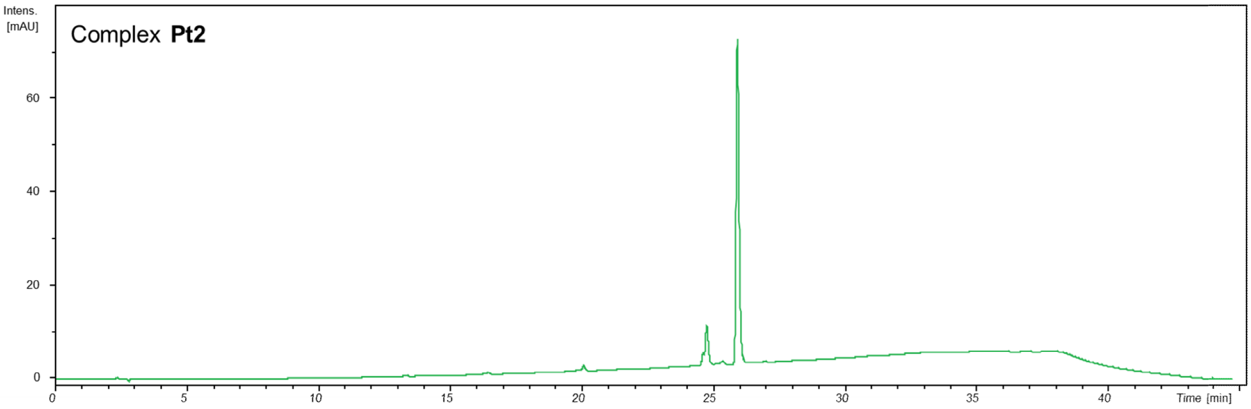

Figure S12. HPLC UV-vis chromatogram of **Pt2** (detection wavelength 254 nm), gradient 1.

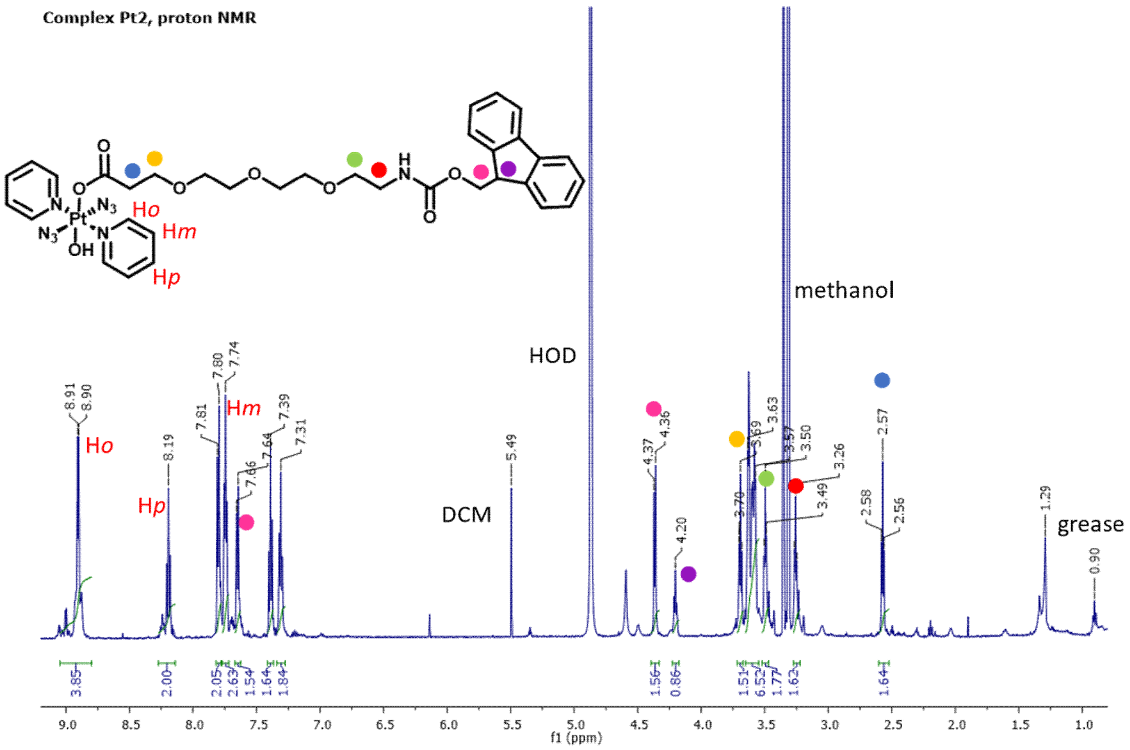

Figure S13. ^{1}H NMR spectrum of complex **Pt2** in CD_3OD.

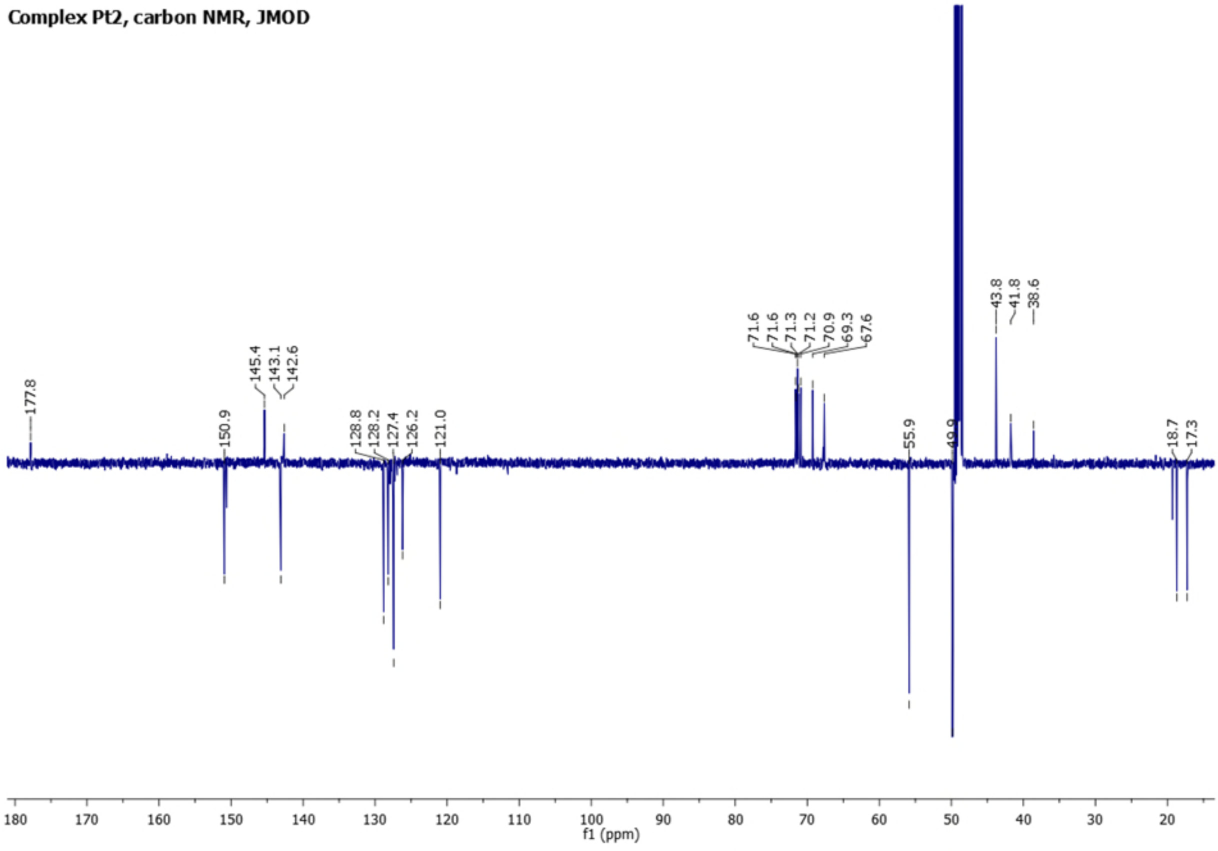

Figure S14. ^{13}C J-modulated spin-echo NMR spectrum of complex **Pt2** in CD_3OD.

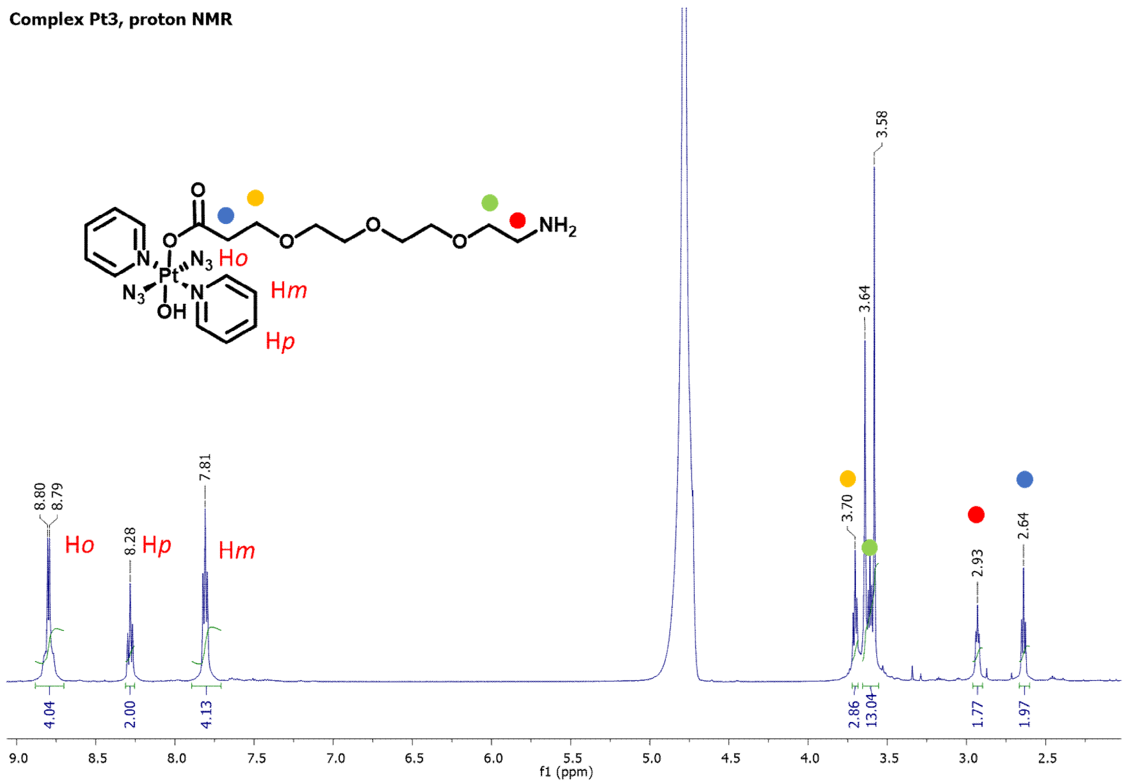

Figure S15. ^{1}H NMR spectrum of complex **Pt3** in D_2O.

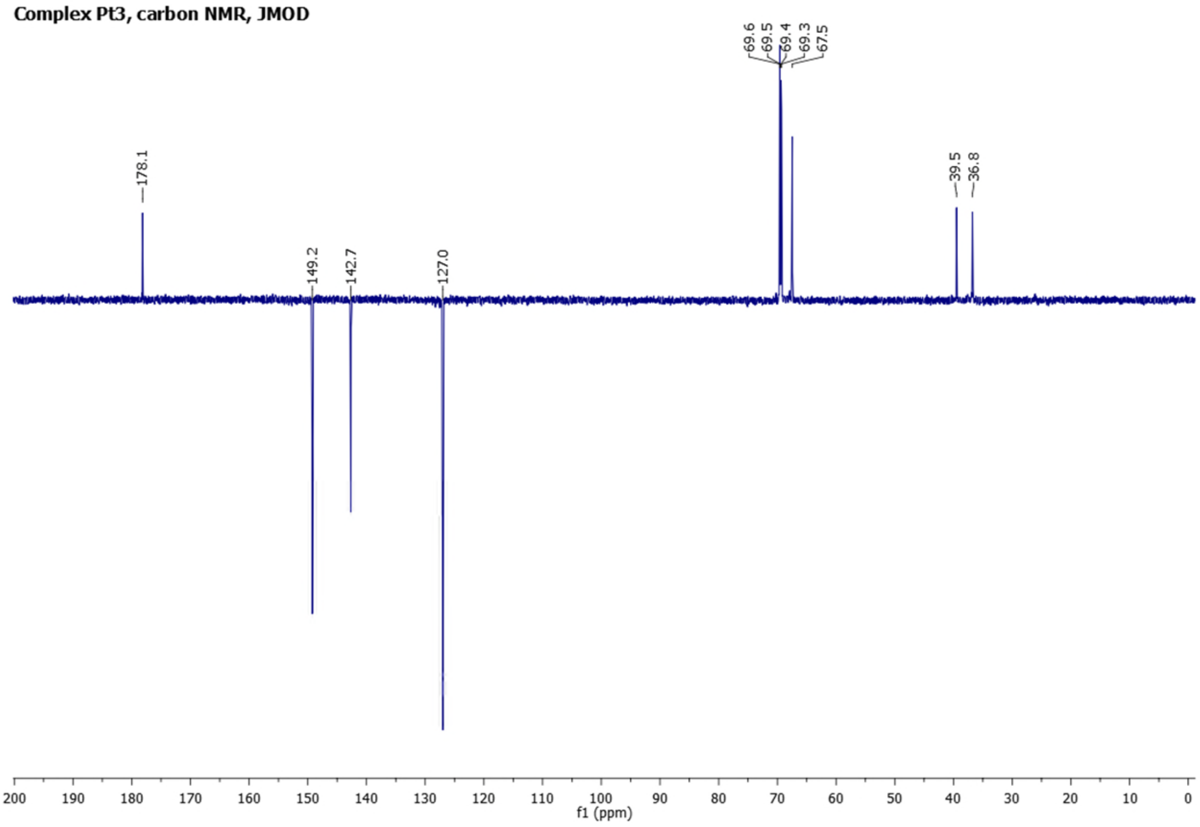

Figure S16. ^{13}C J-modulated spin-echo NMR spectrum of **Pt3** in D_2O.

Complex Pt4, proton NMR

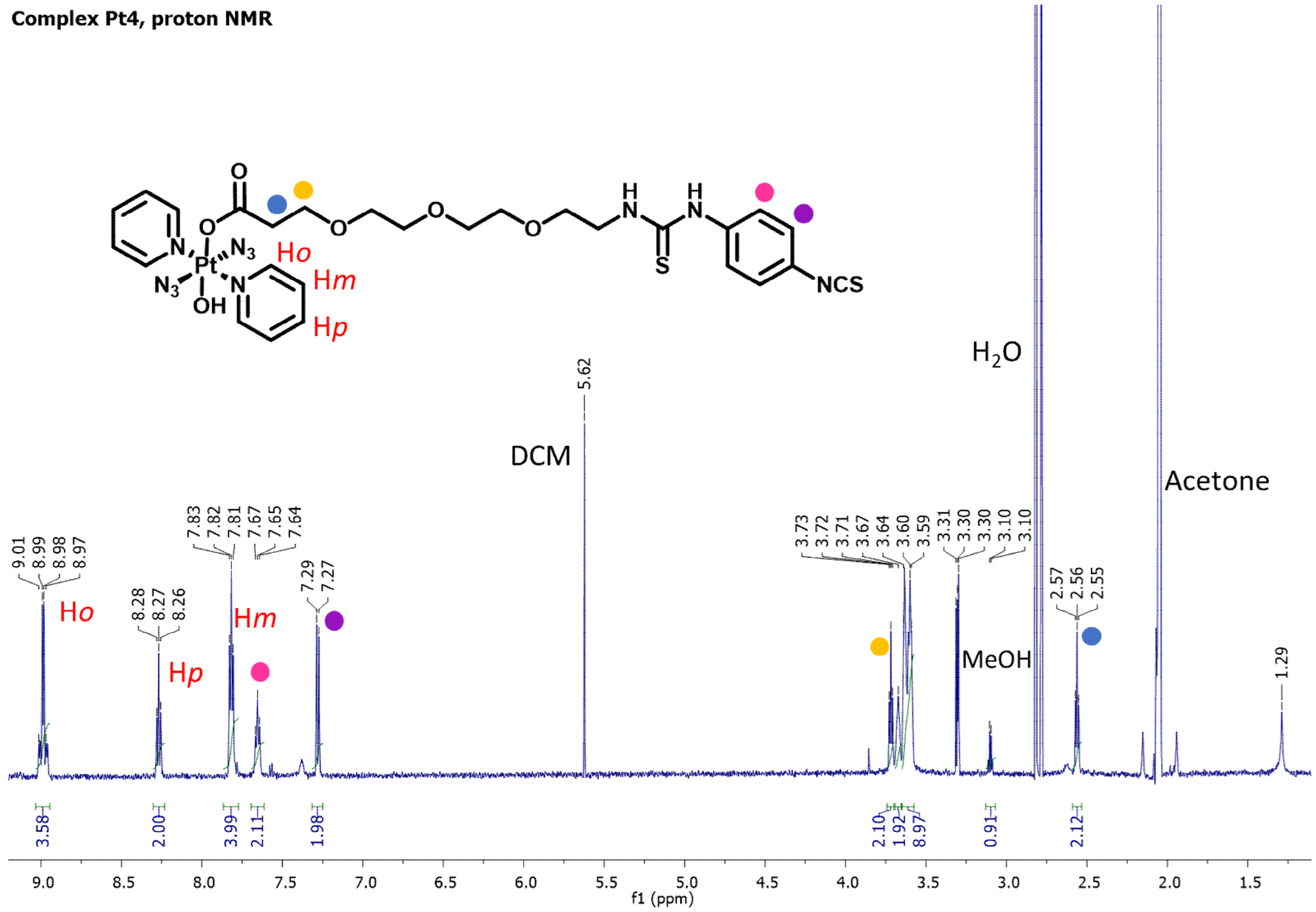

Figure S17. ^{1}H NMR spectrum of complex **Pt4** in acetone d-6.

References

1. N. J. Farrer, J. A. Woods, L. Salassa, Y. Zhao, K. S. Robinson, G. Clarkson, F. S. Mackay and P. J. Sadler, *Angew. Chem.-Int. Ed.*, 2010, **49**, 8905-8908.

2. S. C. Harrison, Blout E. R., *J. Biol. Chem*, 1965, **240**, 209-303
